# Predictors of Postpartum Hemorrhage Following Repeat Cesarean Section: A Retrospective Cohort Study

**DOI:** 10.7759/cureus.109926

**Published:** 2026-05-30

**Authors:** Preeti Banerjee, Maria Sgouraki, Subhashree Nayak, Heena Tiwari, Jasmine Kalsi, Afroz K Syed

**Affiliations:** 1 Department of Obstetrics and Gynecology, Deccan College of Medical Sciences, Hyderabad, IND; 2 Department of Anesthesiology, Dr. Sulaiman Al Habib Hospital, Dubai, SAU; 3 Department of Obstetrics and Gynecology, Government Medical College and Hospital, Sundargarh, IND; 4 Department of Blood Cell, Commissionerate of Health and Family Welfare, Government of Telangana, Hyderabad, IND; 5 Department of General Surgery, Kalpana Chawla Government Medical College and Hospital, Karnal, IND; 6 Department of Dentistry, Anee Yarlagadda Charitable Trust, Vijayawada, IND

**Keywords:** cesarean section, maternal morbidity, placenta previa, postpartum hemorrhage, risk factors

## Abstract

Introduction: Postpartum hemorrhage (PPH) is a major obstetric emergency that contributes substantially to maternal morbidity following cesarean delivery. The identification of high-risk patients undergoing repeat cesarean sections may help improve perioperative preparedness and maternal outcomes. The present study aimed to identify predictors of PPH following repeated cesarean sections.

Materials and methods: A retrospective cohort study was conducted in the Department of Obstetrics and Gynecology on 200 women who underwent repeat cesarean section between January 2020 and December 2025. Demographic, obstetric, and intraoperative data were retrieved from archived medical records. PPH was defined as blood loss ≥1000 mL during or within 24 hours after cesarean delivery or the requirement for transfusion/intervention. Statistical analysis was performed using univariate analysis and multivariable logistic regression to identify independent predictors of PPH. Adjusted odds ratios (aORs) with 95% confidence intervals were calculated, and a p-value < 0.05 was considered statistically significant.

Results: Among the 200 patients, PPH was observed in 72 (36.0%), while 128 (64.0%) did not develop hemorrhage. Women with PPH demonstrated significantly higher body mass index (BMI) (28.3 ± 3.9 kg/m² vs. 25.9 ± 3.3 kg/m²), a greater number of previous cesarean sections (2.1 ± 0.8 vs. 1.6 ± 0.6), lower antenatal hemoglobin levels (9.6 ± 1.4 g/dL vs. 10.7 ± 1.4 g/dL), and prolonged surgery duration (74.8 ± 19.6 min vs. 55.2 ± 14.3 min) (p < 0.001). Placenta previa or placental abnormalities, intraoperative adhesions, and emergency cesarean sections were significantly associated with hemorrhage. Multivariable logistic regression identified placenta previa or placental abnormality (aOR = 3.33), intraoperative adhesions (aOR = 2.50), ≥3 previous cesarean sections (aOR = 2.00), emergency cesarean section (aOR = 1.80), higher parity, elevated BMI, prolonged surgery duration, and lower antenatal hemoglobin level as independent predictors of PPH (all p < 0.05).

Conclusion: PPH following repeated cesarean sections is strongly associated with multiple maternal and intraoperative risk factors. Early identification of high-risk patients may facilitate improved surgical planning, preventive interventions, and optimized maternal care.

## Introduction

Postpartum hemorrhage (PPH) is a leading cause of maternal morbidity and mortality worldwide, particularly in women undergoing cesarean delivery [1]. The increasing global rate of cesarean sections has contributed to a parallel rise in obstetric complications associated with repeated cesarean procedures, including excessive intraoperative and postoperative bleeding [2]. Women with previous cesarean sections are at a higher risk of abnormal placentation, pelvic adhesions, prolonged operative time, and uterine scar-related complications, all of which may predispose them to severe hemorrhage [3]. Despite advances in surgical techniques, anesthetic care, and blood transfusion services, PPH remains a major clinical challenge in tertiary obstetric practice.

Identifying patients at an increased risk of hemorrhage before surgery is essential for optimizing maternal outcomes and reducing preventable complications [3]. Several maternal and obstetric factors, such as obesity, multiparity, anemia, placenta previa, hypertensive disorders, emergency cesarean delivery, and multiple previous cesarean sections, have been reported as potential contributors to PPH [4,5]. However, the relative impact of these predictors may vary between populations and health care settings. Understanding the independent determinants of hemorrhage in women undergoing repeat cesarean sections can aid clinicians in early risk stratification, perioperative planning, resource allocation, and the implementation of preventive strategies.

Recent advances in retrospective clinical data analysis and multivariable regression modeling have enabled the more accurate identification of predictors associated with adverse maternal outcomes. This study aimed to identify the independent predictors of PPH following repeat cesarean sections. The objectives were to assess the association between demographic and clinical variables and PPH, compare perioperative characteristics between patients with and without hemorrhage, and determine significant independent risk factors using multivariate logistic regression analysis.

## Materials and methods

Study design and setting

This retrospective cohort study was conducted in the Department of Obstetrics and Gynecology, Deccan College of Medical Sciences, Hyderabad, India. The study was conducted using archived electronic medical records and hospital case files obtained from an institutional database. Records of patients treated between January 2020 and December 2025 were reviewed. Ethical approval (IEC No: 2026/98/021) was obtained from the ethics committee of the college prior to the commencement of the study, and confidentiality of patient information was strictly maintained throughout the research process. The present retrospective observational study was conducted and reported in accordance with the Strengthening the Reporting of Observational Studies in Epidemiology guidelines to ensure transparent and standardized reporting of observational research.

Study population and sample size

The study population included women with a history of at least one cesarean section who subsequently underwent repeat cesarean delivery during the study period. Sample size estimation was performed using G*Power software version 3.1.9.7 (Heinrich Heine University, Düsseldorf, Germany). Based on previously reported associations between obstetric risk factors and PPH, a moderate effect size of 0.30, a statistical power of 80%, and a 95% confidence interval (CI) with α = 0.05 were considered [6]. The minimum estimated sample size was 180 participants. To improve the reliability of the regression analysis and compensate for incomplete records, 200 eligible patient records were included using consecutive sampling.

Inclusion and exclusion criteria

Women aged ≥18 years who underwent elective or emergency repeat cesarean section after 28 weeks of gestation and had complete perioperative and postoperative records were included in the study. Both singleton and multiple pregnancies were considered eligible for inclusion. Patients with known coagulation disorders, uterine rupture before surgery, incomplete medical records, or cesarean hysterectomy performed for non-obstetric gynecological indications were excluded from the study.

Data collection procedure

Data extraction was performed using a structured data collection form developed specifically for the study to ensure consistency of retrospective record review. Information was retrieved from archived electronic medical records and operative notes available within the institutional database. Baseline demographic, maternal, obstetric, and intraoperative variables were recorded from the patient files and electronic medical records. The variables assessed included maternal age, body mass index (BMI), gravidity, parity, number of previous cesarean sections, gestational age at delivery, antenatal hemoglobin level, placenta previa or placental abnormalities, hypertensive disorders of pregnancy, diabetes mellitus, multiple pregnancies, type of cesarean section (elective or emergency), duration of surgery, and the presence of intraoperative adhesions.

Outcome assessment

The primary outcome assessed in the present study was PPH following repeat cesarean section. PPH was defined as an estimated blood loss of ≥1000 mL during surgery or within 24 hours after cesarean delivery, a requirement for blood transfusion, or a need for pharmacological or surgical intervention to control hemorrhage, according to institutional obstetric management protocols [7].

Statistical analysis

Statistical analysis was performed using IBM SPSS Statistics for Windows, Version 26.0 (Released 2019; IBM Corp., Armonk, New York). Continuous variables were expressed as mean ± standard deviation, whereas categorical variables were presented as frequencies and percentages. Normality of distribution was assessed using the Shapiro-Wilk test. The independent-samples t-test or Mann-Whitney U test was used to compare continuous variables depending on data distribution. The chi-squared test, or Fisher’s exact test, was used for categorical variables. Variables demonstrating statistical significance in the univariate analysis were further entered into the multivariable logistic regression analysis to identify independent predictors of PPH. Adjusted odds ratios (aOR) with 95% CI were also calculated. Statistical significance was set at p < 0.05.

## Results

A total of 200 women underwent repeat cesarean section during the study period. PPH was observed in 72 patients (36.0%), whereas 128 patients (64.0%) did not develop hemorrhage. Table 1 presents the univariate analysis of continuous variables associated with PPH. Women with PPH showed significantly higher BMI, gravidity, parity, and number of previous cesarean sections than those without PPH (p < 0.05). Lower gestational age and reduced antenatal hemoglobin levels were significantly associated with hemorrhage. The duration of surgery was also significantly prolonged in the women with PPH (p < 0.001). Maternal age did not demonstrate a statistically significant association with PPH (p = 0.144).

**Table 1:** Univariate analysis of continuous variables associated with postpartum hemorrhage (PPH) following repeat cesarean section. Data were presented as mean ± standard deviation (SD). An independent-samples t-test was used for normally distributed continuous variables, and the Mann-Whitney U test was used for non-normally distributed variables. A *p-value < 0.05 was considered statistically significant. U: Mann-Whitney U statistic.

Continuous variables	PPH (n = 72) Mean ± SD	No PPH (n = 128) Mean ± SD	Test value	p-value
Maternal age (years)	29.1 ± 4.9	28.0 ± 4.4	t = 1.47	0.144
BMI (kg/m²)	28.3 ± 3.9	25.9 ± 3.3	t = 4.29	< 0.001*
Gravidity	3.4 ± 1.3	2.9 ± 1.1	t = 2.58	0.011*
Parity	2.7 ± 1.1	2.2 ± 0.9	t = 3.01	0.003*
Number of previous cesarean sections	2.1 ± 0.8	1.6 ± 0.6	t = 4.62	< 0.001*
Gestational age (weeks)	37.2 ± 2.1	37.9 ± 1.6	t = −2.31	0.022*
Antenatal hemoglobin (g/dL)	9.6 ± 1.4	10.7 ± 1.4	t = −4.89	< 0.001*
Duration of surgery (min)	74.8 ± 19.6	55.2 ± 14.3	U = 2483	< 0.001*

Table 2 summarizes the univariate analysis of the categorical variables associated with PPH. Placenta previa or placental abnormalities were identified in 24 women (63.2%) with PPH and 14 women (36.8%) without PPH, demonstrating a significant association with hemorrhage (p < 0.001). Intraoperative adhesions were present in 34 women (56.7%) with PPH and 26 women (43.3%) without PPH (p < 0.001). An emergency cesarean section was performed in 38 patients (45.2%) with PPH and 46 (54.8%) patients without PPH, showing a statistically significant association (p = 0.029). Hypertensive disorders, diabetes mellitus, and multiple pregnancies were not significantly associated with hemorrhage (p > 0.05).

**Table 2:** Univariate analysis of categorical variables associated with PPH following repeat cesarean section. Data were presented as frequency and percentage. The chi-square test was used for the comparison of categorical variables; *p-value < 0.05 was considered statistically significant. χ²: chi-square statistic; CS: cesarean section.

Categorical variables	PPH (n = 72) n (%)	No PPH (n = 128) n (%)	Test value	p-value
Placenta previa/abnormality	24 (63.2)	14 (36.8)	χ² = 18.43	< 0.001*
Hypertensive disorders	26 (48.1)	28 (51.9)	χ² = 3.12	0.077
Diabetes mellitus	18 (42.9)	24 (57.1)	χ² = 0.89	0.346
Multiple pregnancy	11 (50.0)	11 (50.0)	χ² = 2.44	0.118
Intraoperative adhesions	34 (56.7)	26 (43.3)	χ² = 12.68	< 0.001*
Emergency CS (vs. elective)	38 (45.2)	46 (54.8)	χ² = 4.78	0.029*

Table 3 presents the results of multivariable logistic regression analysis to identify the independent predictors of PPH. Higher BMI, ≥3 previous cesarean sections, prolonged duration of surgery, placenta previa or placental abnormalities, intraoperative adhesions, emergency cesarean section, and higher parity were independently associated with an increased risk of PPH (p < 0.05). Lower antenatal hemoglobin levels were independently associated with higher odds of hemorrhage. Placenta previa or placental abnormality demonstrated the strongest association with PPH (aOR = 3.33; 95% CI: 1.79-6.22; p < 0.001). The regression model demonstrated satisfactory goodness of fit, with a Nagelkerke R² value of 0.412 and an overall prediction accuracy of 79.5%.

**Table 3:** Multivariable logistic regression analysis of independent predictors of PPH following repeat cesarean section. Variables significant in univariate analysis (p < 0.05) were entered into multivariable logistic regression using the Enter method. Model fit statistics: Nagelkerke R² = 0.412, Hosmer-Lemeshow χ² = 6.24, p = 0.621, and overall accuracy = 79.5%. Reference categories: CS type - elective, previous CS - <3 sections; *p < 0.05 statistically significant, model adequately fits data (Hosmer-Lemeshow p = 0.621). B: regression coefficient; SE: standard error; Wald χ²: Wald chi-square statistic; aOR: adjusted odds ratio; CS: cesarean section; df: degree of freedom.

Variable	B	SE	Wald χ²	df	p-value	aOR (95% CI)
BMI (kg/m²)	0.218	0.062	12.34	1	< 0.001*	1.24 (1.10-1.41)
Number of previous CS (≥3 vs. <3)	0.693	0.201	11.87	1	0.001*	2.00 (1.35-2.96)
Antenatal hemoglobin (g/dL)	−0.382	0.098	15.21	1	< 0.001*	0.68 (0.56-0.83)
Duration of surgery (min)	0.041	0.011	13.74	1	< 0.001*	1.04 (1.02-1.06)
Placenta previa/abnormality	1.204	0.318	14.31	1	< 0.001*	3.33 (1.79-6.22)
Intraoperative adhesions	0.916	0.264	12.02	1	0.001*	2.50 (1.49-4.20)
Emergency CS	0.588	0.235	6.26	1	0.012*	1.80 (1.14-2.85)
Parity	0.314	0.148	4.50	1	0.034*	1.37 (1.03-1.83)

## Discussion

PPH remains a major contributor to maternal morbidity following cesarean delivery, particularly in women undergoing repeat cesarean sections. This retrospective cohort study identified several significant maternal, obstetric, and intraoperative predictors associated with PPH, including higher BMI, increased parity, multiple previous cesarean sections, low antenatal hemoglobin levels, prolonged duration of surgery, placenta previa or placental abnormalities, intraoperative adhesions, and emergency cesarean section. These findings emphasize the multifactorial nature of hemorrhagic complications in repeated cesarean deliveries and highlight the importance of early risk stratification and perioperative preparedness.

In the present study, higher BMI was independently associated with an increased risk of PPH. Obesity increases technical surgical difficulty, prolongs the operative duration, and impairs uterine contractility, thereby contributing to excessive blood loss. Increased adipose tissue may also lead to reduced visualization during surgery and higher rates of operative complications. Similar findings have been reported in previous studies, which showed that maternal obesity significantly increased the risk of obstetric hemorrhage during cesarean delivery [6,8,9]. The association between elevated BMI and PPH further underscores the importance of antenatal weight management and careful intraoperative monitoring in obese pregnant women.

The number of previous cesarean sections was another significant predictor in this study. Women with three or more previous cesarean sections had substantially higher odds of hemorrhage. Repeated uterine surgeries increase scar formation, pelvic adhesions, and abnormal placentation, all of which contribute to difficult surgical dissection and increase intraoperative bleeding [10]. Similar observations were documented by Silver et al. [11], who demonstrated that maternal morbidity progressively increases with an increasing number of cesarean deliveries. Placenta previa and placenta accreta spectrum disorders are also more common in women with multiple prior cesarean sections, further increasing the risk of hemorrhage.

Lower antenatal hemoglobin levels were significantly associated with PPH in the present study. Maternal anemia reduces physiological reserves and increases the susceptibility to adverse outcomes following blood loss. Women with pre-existing anemia may experience earlier hemodynamic instability, even with moderate hemorrhage [12]. Previous studies have also identified antenatal anemia as an important predictor of severe obstetric hemorrhage [13,14]. Therefore, optimizing maternal hemoglobin levels during antenatal care through nutritional supplementation, iron therapy, and the early detection of anemia may play a crucial role in reducing maternal complications.

In this study, placenta previa and placental abnormalities were strongly associated with PPH. Abnormal placentation is a well-established risk factor for severe hemorrhage due to defective placental separation and increased vascularity at the implantation site [15]. Surgical management of placenta previa often requires extensive hemostatic interventions and blood transfusions. Similar findings were reported by Jauniaux et al. [16], who emphasized that placenta previa and placenta accreta spectrum disorders were among the most significant contributors to massive obstetric hemorrhage during cesarean delivery. Early antenatal diagnosis using ultrasonography and multidisciplinary surgical planning is essential for high-risk cases.

Intraoperative adhesions and prolonged surgical duration were also identified as independent predictors of hemorrhage. Dense pelvic adhesions resulting from previous surgeries may increase operative difficulty, prolong tissue dissection, and predispose patients to vascular injury [16]. A prolonged operative time may reflect technically challenging procedures associated with greater blood loss. Comparable findings have been reported in studies evaluating repeat cesarean delivery outcomes, where extensive adhesions are associated with increased maternal morbidity and operative complications [3-5].

In this study, emergency cesarean section was significantly associated with an increased risk of PPH. Emergency procedures are often performed under urgent clinical circumstances with limited time for adequate preoperative optimization. Conditions such as fetal distress, obstructed labor, placental complications, and uterine atony may further predispose patients to hemorrhage. Previous investigations by Magann et al. [17] and Sheldon et al. [18] demonstrated higher rates of maternal complications and blood loss in emergency cesarean deliveries than in elective procedures.

The findings of this study have several important clinical implications. Identification of high-risk patients prior to surgery can facilitate individualized obstetric management, availability of blood products, experienced surgical teams, and implementation of preventive strategies such as active management of the third stage of labor and prophylactic uterotonic administration. The development of institutional risk assessment protocols based on the identified predictors may improve maternal safety and reduce hemorrhage-related morbidity.

Strengths of the present study include the focus on women undergoing repeat cesarean section, an increasingly important high-risk obstetric population. The sample size was estimated a priori using G*Power software version 3.1.9.7 (Heinrich Heine University, Düsseldorf, Germany), and consecutive sampling was employed to reduce potential selection bias. The study incorporated both demographic and intraoperative variables, allowing a more comprehensive evaluation of factors associated with PPH. In addition, multivariable logistic regression analysis with model fit assessment using Nagelkerke R² and Hosmer-Lemeshow testing strengthened the analytical rigor and reliability of the findings. The use of real-world tertiary care clinical data further enhances the practical relevance of the study findings in routine obstetric practice.

Certain limitations of this study should be considered when interpreting its findings. The retrospective design relied on the accuracy and completeness of the medical records, which may have introduced an information bias. This study was conducted at a single tertiary care center, which limits its generalizability to other healthcare settings and populations. Additionally, some potentially relevant variables, such as estimated intraoperative blood loss techniques, surgeon experience, and postoperative management protocols, were not uniformly available in all the records. Quantitative blood loss measurement methods were also not routinely available, and hemorrhage assessment was based primarily on documented clinical estimates and interventions, which may have introduced variability in outcome classification. Furthermore, although multivariable logistic regression identified significant independent predictors of PPH, the study was not designed to develop or validate a formal predictive model or risk stratification tool. Advanced predictive performance analyses such as ROC curve analysis and external validation were beyond the scope of the present study and should be explored in future large-scale prospective multicenter investigations. Despite these limitations, this study provides valuable insights into the clinically relevant predictors of PPH following repeat cesarean sections and may assist in improving risk-based obstetric care.

## Conclusions

PPH following repeat cesarean section is associated with multiple maternal, obstetric, and intraoperative factors. The present study identified higher BMI, multiple previous cesarean sections, lower antenatal hemoglobin levels, placenta previa or placental abnormalities, intraoperative adhesions, emergency cesarean section, higher parity, and prolonged operative duration as significant factors associated with increased hemorrhage risk. Among these, placenta previa demonstrated the strongest association with PPH. Although the study was not designed to develop a formal predictive model, the identified factors may assist clinicians in recognizing high-risk patients requiring enhanced perioperative preparedness and monitoring. Early risk recognition and individualized obstetric management may contribute to improved surgical planning, timely preventive interventions, and optimization of maternal outcomes in women undergoing repeat cesarean delivery.
